# Nanoencapsulated Curcumin: Enhanced Efficacy in Reversing Memory Loss in An Alzheimer Disease Model

**DOI:** 10.3390/brainsci14020130

**Published:** 2024-01-26

**Authors:** Anne Suély Pinto Savall, Jhuly Dorneles de Mello, Eduarda Monteiro Fidelis, Antonio Alvenir Comis-Neto, Maria Regina Nepomuceno, Camila de Oliveira Pacheco, Sandra Elisa Haas, Simone Pinton

**Affiliations:** 1 Research Group on Biochemistry and Toxicology in Eukaryotes, Federal University of Pampa, Campus Uruguaiana, Uruguaiana 97500-970, RS, Brazil; annesavall@gmail.com (A.S.P.S.); jhulymello.aluno@unipampa.edu.br (J.D.d.M.); eduardamonfil.aqua@gmail.com (E.M.F.); antoniocomis.aluno@unipampa.edu.br (A.A.C.-N.); marianepomuceno.aluno@unipampa.edu.br (M.R.N.); 2 Laboratory of Pharmacology and Pharmacometrics, Federal University of Pampa, Campus Uruguaiana, Uruguaiana 97500-970, RS, Brazil; coliveirapacheco@gmail.com (C.d.O.P.); sandrahaas@unipampa.edu.br (S.E.H.)

**Keywords:** memory, streptozotocin, nanotechnology, neuroinflammation, oxidative stress

## Abstract

Investigating new drugs or formulations that target Alzheimer disease (AD) is critical for advancing therapeutic interventions. Therefore, this study aimed to assess the effectiveness of nanoencapsulated curcumin (NC Curc) in alleviating memory impairment, oxidative stress, and neuroinflammation in a validated AD model. Male Wistar rats were given bilateral intracerebroventricular injections of either saline or streptozotocin (STZ) (3 mg/3 µL/site) to establish the AD model (day 0). On day 22, daily oral administrations of curcumin (6 mg/kg), NC Curc (6 mg/kg), or a vehicle (unloaded NC) were initiated and continued for 14 days. NC Curc significantly reversed memory deficits in object recognition and inhibitory avoidance tests induced by STZ. Both formulations of curcumin attenuated elevated acetylcholinesterase activity caused by STZ. Importantly, NC Curc alone effectively mitigated STZ-induced oxidative stress. Additionally, NC Curc treatment normalized GFAP levels, suggesting a potential reduction in neuroinflammation in STZ-treated rats. Our findings indicate that NC Curc improves memory in an AD rat model, highlighting its enhanced therapeutic effects compared to unencapsulated curcumin. This research significantly contributes to understanding the therapeutic and neurorestorative potential of NC Curc in AD, particularly in reversing pathophysiological changes.

## 1. Introduction

Alzheimer disease (AD) is a complex condition influenced by various factors, including genetics, environment, and metabolism. Its main characteristic is memory loss, which is the first indication of dementia. The disease is characterized by the buildup of β-amyloid plaques, which initiate harmful effects on the brain, causing the loss of neurons and synapses. This process also increases oxidative stress and inflammation, damaging the neural tissue. As a result, cognitive decline worsens over time, particularly affecting brain regions such as the cortex, hippocampus, striatum, and cerebellum [1,2,3].

The complex pathophysiology of AD involves oxidative stress and neuroinflammation. These factors adversely affect the central nervous system, disrupting metabolic pathways in the brain and causing irreversible damage to the biological system. Oxidative stress plays a crucial role in the various stages of AD, including both sporadic and familial forms [1,4]. Neuroinflammation is a key factor in the progression of AD, as it complicates the brain environment and impairs neuronal and synaptic functions [5]. AD patients also experience a progressive cognitive decline associated with changes in acetylcholine levels [6]. Maintaining optimal brain function requires regulating oxidative stress, neuroinflammation, and acetylcholine levels, especially in the cortex and hippocampus [5,7]. Low molecular weight antioxidants and anti-inflammatory agents, such as *Curcuma longa* L., can help counteract the brain changes seen in AD patients [4]. *C. longa* L., an herbaceous plant of the Zingiberaceae family, receives its distinctive yellow-orange color from curcumin, its primary curcuminoid [8].

Curcumin has garnered attention for its potential therapeutic benefits in neurodegenerative conditions, specifically AD [4]. It is believed to work by inhibiting the formation and promoting the disintegration of β-amyloid plaques, as well as mitigating the hyperphosphorylation of the tau protein [9]. One limitation of curcumin is its low solubility and permeability in water, resulting in low bioavailability and presenting challenges for oral administration [10]. However, by utilizing nanocarrier systems, particularly nanocapsules, it becomes possible to achieve precise drug targeting to specific tissues [11]. This approach enhances drug permeability across physiological barriers, such as the blood–brain barrier, optimizing biodistribution and potentially enhancing the therapeutic efficacy of curcumin [12,13,14,15]. 

Considering these factors, the objective of this study was to evaluate the effect of curcumin nanocapsules (NC Curc) on memory loss, neuroinflammation, and oxidative stress reversal in an animal model of AD induced by intracerebroventricular (icv) injection of streptozotocin (2-deoxy-2-(3-(methyl-3-nitrosoureido)-d-glucopyranose) (STZ). Additionally, this study investigated the involvement of acetylcholinesterase (AChE) activity. To mimic the sporadic non-hereditary form of AD, which is the most common category affecting patients and is related to interactions between genetic and environmental factors [16], low doses of STZ were used to induce this well-established model through an icv injection capable of inducing a pathology similar to AD [3,16].

## 2. Materials and Methods

### 2.1. Animals

A total of 86 male 8-week-old Wistar rats, weighing 250–350 g, were employed in this study: 62 rats for behavioral, oxidant, and AChE assays, with an additional set of 24 rats (*n* = 4/group) for immunohistochemistry assays. The animals were housed at a controlled temperature of 22–25 °C, with unrestricted access to water and food. They were subjected to a 12 h:12 h light/dark cycle, with lights turning on at 7:00 a.m. The rats were sourced from the Federal University of Santa Maria (Brazil) and used in accordance with the guidelines of the Committee on Care and Use of Experimental Animal Resources of the Federal University of Pampa (Brazil; protocol no. 040/2019). The sample size was based on similar studies [17,18]. All efforts were focused on minimizing animal suffering and reducing the number of animals used in the experiments.

### 2.2. Drugs and Formulations

Streptozotocin was obtained from Sigma-Aldrich (S0130) (St. Louis, MO, USA) and dissolved in Hank’s balanced salt solution (HBSS) with the following concentrations (in mM): 137 NaCl, 0.63 Na_2_HPO_4_, 4.17 NaHCO_3_, 5.36 KCl, 0.44 KH_2_PO_4_, 1.26 CaCl_2_, 0.41 MgSO_4_, 0.49 MgCl_2_, and 10 glucose (pH 7.4), as instructed by Biasibetti et al. [19]. Curcumin was obtained from Sigma-Aldrich (C1386) (St. Louis, MO, USA). The anti-glial fibrillary acidic protein (anti-GFAP) (G3893) antibody was obtained from Sigma-Aldrich Brazil, along with a secondary antibody labeled with Alexa Fluor 594 from Invitrogen (A11012). DAPI fluorescent dye was sourced from Invitrogen (D9542). All other chemicals were of analytical grade and procured from standard commercial suppliers.

Curcumin nanocapsules coated with polysorbate (P80) formulation were prepared using the deposition interfacial method [20]. Curc (final concentration of 0.6 mg/mL), poly(ε-caprolactone), caprylic/capric triglyceride oil, and sorbitan monostearate were dissolved in acetone and then poured into an aqueous solution containing P80. After agitation, a rotatory evaporator was used to remove the organic solvent and excess water. Unloaded NCs were prepared using the same method without curcumin. Formulations were characterized by Santos et al., 2021 [20] and showed particle sizes of 254 ± 2 and 207 ± 5 nm for NC Curc and unloaded NCs, respectively. The zeta potential and pH for NC Curc were −26 ± 2 mV and 6.0 ± 0.01 and, for unloaded NC, were −25 ± 3 mV and 5.0 ± 0.01. Encapsulation efficiency and drug content were close to 100% for NC Curc.

### 2.3. Surgical Procedure and Experimental Protocol

The initial day involved icv infusion of STZ or vehicle, as demonstrated elsewhere [17,19]. Specifically, on the day of surgery, animals were anesthetized using ketamine/xylazine (75 and 10 mg/kg, respectively, ip) and positioned in a stereotaxic apparatus. A midline sagittal incision was made in the scalp, and burr holes drilled over the lateral ventricles. The coordinates used were 0.9 mm posterior to bregma, 1.5 mm lateral to sagittal suture, and 3.6 mm beneath the brain surface. Rats received 3 μL bilateral infusion of STZ (3 mg/kg) or vehicle using a Hamilton syringe. Post-surgery, rats were placed on a heating pad at 37.5 ± 0.5 °C until recovery from anesthesia. Subsequently, they were housed for 21 days to establish the AD model, after which they were redivided into groups to assess NC Curc and curcumin’s effects on STZ. The animals were assigned to six groups (*n* = 9–12/group):

(I). Control—vehicle (HBSS, 3 μL/ventricule, icv) + unloaded NC (gavage) (*n* = 12)
(II). Curc—vehicle (HBSS, 3 μL/ventricule, icv) + free curcumin (in canola oil, 6 mg/kg, gavage) (*n* = 11)
(III). NC Curc—vehicle (HBSS, 3 μL/ventricule, icv) + curcumin-loaded NC (6 mg/kg, gavage) (*n* = 12)
(IV). STZ—streptozotocin (3 mg/kg, 3 μL/ventricule, icv) + unloaded NC (gavage) (*n* = 9)
(V). STZ + Curc—streptozotocin (3 mg/kg, 3 μL/ventricule, icv) + free curcumin (in canola oil, 6 mg/kg, gavage) (*n* = 9)
(VI). STZ + NC Curc—streptozotocin (3 mg/kg, icv) + curcumin-loaded NC (6 mg/kg, gavage) (*n* = 9)

**Figure 1:**
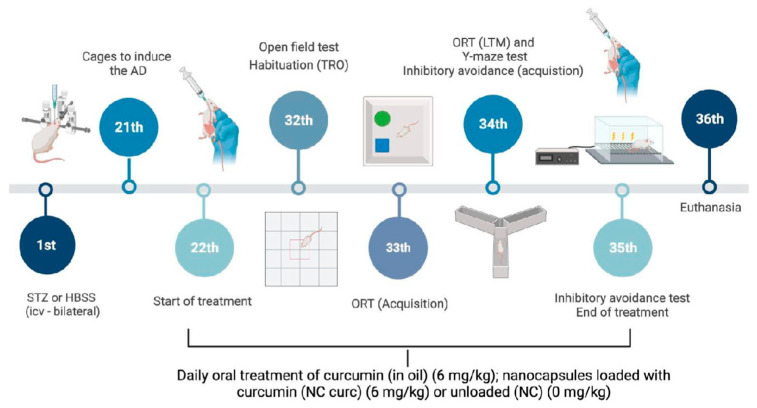
Schematic representation of the experimental design. On day 0, rats received a bilateral infusion of STZ (3 mg/kg—3 μL) or saline solution intracerebroventricular (icv). After 21 days, the rats were divided into groups. From day 22 to day 35, the animals were treated with free curcumin (Curc) (in oil, 6 mg/kg, ig), curcumin loaded in nanocapsules (NC Curc) (6 mg/kg, ig), or unloaded nanocapsules (NC) (0 mg/kg). Behavioral tests were carried out between days 32 and 35 to assess cognitive function. On day 36, the prefrontal cortices and hippocampi were dissected.

On day 22, the rats began daily oral treatment, continued for 14 days, with curcumin in oil (10 mL/kg), NC Curc (6 mg/kg), or unloaded NC (0 mg/kg). The dosages were selected based on the study by Loch-Neckel et al. [21]. Behavioral tests were conducted on days 32–35. Twenty-four hours after the last behavior test, on day 36, the rats were euthanized, and their prefrontal cortices and hippocampi were dissected. A schematic representation of the experimental procedure is shown in Figure 1.

### 2.4. Behavioral Tests

#### 2.4.1. Open Field Test

On day 32, spontaneous locomotor activity was assessed using the open field test [22]. The apparatus, measuring 50 × 50 × 50 cm, had its floor divided into nine equal squares. Each rat was placed in the center, and their movement across segments (four-leg criterion) and rearings were recorded for 5 min by a blinded observer using a stopwatch.

#### 2.4.2. Object Recognition Test

Long-term memory (LTM) was measured using the one-trial object recognition task (ORT) method on days 32, 33, and 34 following icv STZ administration [23,24]. The animals were placed in an open wooden box (50 × 50 × 50 cm) and allowed to explore two identical objects (A1 and A2) for 5 min on the training day (day 33). The objects, made of plastic with various colors, were attached to the floor to prevent displacement. To measure LTM, one of the objects was replaced with a novel object (B) 24 h after training, and the exploration time was measured by a blinded observer for an additional 5-min period. Exploration was defined as sniffing or touching the object with the nose and/or forepaws. The results were expressed as the percentage of exploratory preference for each animal using the ratio tB/(tA + tB) × 100, where tA is the time spent exploring the familiar object A and tB is the time spent exploring the novel object B.

#### 2.4.3. Y-Maze Test

Spatial working memory was assessed on day 34 after icv STZ administration for 5 min using a Y-maze, as described by Dellu et al. [25] and then validated as a task requiring hippocampal function and spatial memory [26,27]. The Y-maze used in this study had three arms crossing at 120° angles. This behavioral test was performed by a blinded observer. Rats were expected to explore the new arms more frequently than a recently explored arm, and returning to a previously explored arm was counted as a mistake. A lower exploration frequency of the last previously explored arm indicated better memory performance. A consecutive entry into all three arms (i.e., ABC, CAB, or BCA but not ABA) was considered an actual alternation. The scores were calculated as (actual alternation/maximal alternation − 2) × 100.

#### 2.4.4. Inhibitory Avoidance Test

Aversive LTM was assessed using the inhibitory avoidance test on days 34 and 35 [17]. The apparatus consisted of a single box with a safe platform, and the rats were trained to associate the removal of this platform with an aversive stimulus (a 0.5 mA electric shock delivered for 2 s). Twenty-four hours after training (day 35), the rats were placed on the platform again, and the time taken to step down (transfer latency) was measured (cut-off at 300 s). Latency (in seconds) was defined as the time taken to fall from the platform in the acquisition and retention phases. This behavioral test was performed by a blinded observer.

### 2.5. Sample Preparation

Samples were homogenized in 50 mM Tris-HCl buffer, pH 7.4 (1/10, *w*/*v*). Post-homogenization, samples were centrifuged at 2400 *g* for 10 min at 4 °C. The resultant low-speed supernatant fraction (S1) was utilized for several analyses: reactive species (RS) and non-protein thiol (NPSH) levels, thiobarbituric acid reactive species (TBARS) levels, and catalase (CAT) and acetylcholinesterase (AChE) enzymatic activities. For immunohistochemistry, rats were deeply anesthetized with ketamine/xylazine (ip) and perfused through the left cardiac ventricle first with 0.9% saline solution, then with 4% paraformaldehyde in 0.1 M PBS, pH 7.4. Brains were post-fixed in the same fixative for 24 h at room temperature and cryoprotected in 30% sucrose solution in PBS at 4 °C for 48 h. Finally, blinded observers froze the brains and stored them at −80 °C for future use in assays.

### 2.6. Oxidative Stress Parameters

#### 2.6.1. Reactive Species

Reactive species (RS) levels were measured spectrofluorometrically using 2′,7′-dichlorofluorescein diacetate (DCHF-DA), a non-fluorescent compound that becomes fluorescent dichlorofluorescein (DCF) upon oxidation by RS [28]. S1 was diluted (1:10) in 50 mM Tris-HCl (pH 7.4), mixed with 10 µL of 1 mM DCHF-DA, and incubated at room temperature for 60 min. The fluorescence intensity of DCF was recorded at 520 nm (with 480 nm excitation). RS levels are expressed in units of DCF fluorescence.

#### 2.6.2. Non-Protein Thiol Levels

NPSH levels were assessed using Ellman’s method [29]. S1 was combined with 10% trichloroacetic acid (1:1 ratio). Post-centrifugation, the protein pellet was discarded, and free thiol groups in the supernatant were measured. The supernatant was mixed with 1 M potassium phosphate buffer (pH 7.4) and 10 mM 2-nitro-5-thiobenzoic acid. The colorimetric reaction was quantified at 412 nm, with NPSH levels expressed as nmol NPSH/g tissue.

#### 2.6.3. Thiobarbituric Acid Reactive Species Levels

Lipid peroxidation was determined by TBARS assay as described by Ohkawa et al. [30] by measuring the concentration of malondialdehyde (MDA) as an end product of lipid peroxidation by reaction with thiobarbituric acid (TBA). Tissue aliquot (S1) was incubated at 95 °C for 2 h with TBA. Color reaction was measured at 532 nm. TBARS was reported in nmol TBARS/mg of protein.

#### 2.6.4. Catalase Activity

The CAT activity in S1 was determined spectrometrically using Aebi’s method [31], which measures the disappearance of H_2_O_2_ in the presence of the sample at 240 nm. An aliquot of S1 was added to a 50 mM potassium phosphate buffer (pH 7.0), and the enzymatic reaction was initiated by adding H_2_O_2_. One unit of enzyme was defined as the amount required to monitor the disappearance of H_2_O_2_. Enzymatic activity is expressed as units, which is the amount that decomposes 1 μmol H_2_O_2_/min at pH 7 and 25 °C per mg protein.

### 2.7. Acetylcholinesterase Activity

Prefrontal cortex samples were homogenized in 0.25 M sucrose buffer (1/10, *w*/*v*) and centrifuged at 2400× *g* for 15 min at 4 °C. The resulting low-speed supernatants (S1) were used for the acetylcholinesterase (AChE) assay. AChE activity was determined as per Ellman et al. [32], using acetylthiocholine as a substrate. S1 was incubated with 0.1 M potassium phosphate buffer (pH 7.4) at 25 °C for 2 min, followed by the addition of 50 μL of 10 mM 5,5-dithiobis-(2-nitrobenzoic) acid and 200 μL of 8 mM acetylthiocholine. Spectrophotometric readings were taken at 412 nm every 30 s. AChE activity was expressed as μmol/h/mg protein.

### 2.8. Immunohistochemistry Assay

Coronal brain sections (30 µm) were prepared using a cryostat (Leica-CM 3050S) at −20 °C. Sections were incubated for 60 min in a blocking buffer with 10% normal donkey serum (DS) in PBS with 0.1% Triton X-100 at room temperature. They were then incubated overnight at 4 °C with rabbit anti-GFAP (1:400) in 1% DS diluted in 0.5% PBS-Tx. After washing in PBS, sections were incubated with anti-rabbit Alexa 594 (1:1000) in 1% DS diluted in 0.5% PBS-Tx for 2 h at room temperature. Subsequent to three PBS washes, sections were incubated with 0.5 μg/mL DAPI (Invitrogen) for 10 min. After the final washes, sections were mounted on slides with Fluor Save (Merck) and covered with coverslips. Images of the hippocampal region were captured using the EVOS FLoid Imaging System, with GFAP fluorescence integrated density analyzed using the NIH Image J software (15 November 2023). The method of Pinton et al. [17] was used, with minor modifications [33].

### 2.9. Protein Levels

Protein concentration in brain homogenates was quantified using Bradford’s method [34]. Samples and bovine serum albumin standards were diluted 1:50 in potassium phosphate buffer (10 mM, pH 7.4) and incubated with Bradford reagent for 10 min at room temperature.

### 2.10. Statistical Analysis 

Data were presented as mean ± SEM. Statistical differences between groups were determined using one-way analysis of variance (ANOVA) followed by Tukey’s multiple range test, as appropriate (GraphPad, San Diego, CA, USA). A *p*-value of <0.05 was considered statistically significant. 

## 3. Results

### 3.1. Behavioral Tests

**Figure 2:**
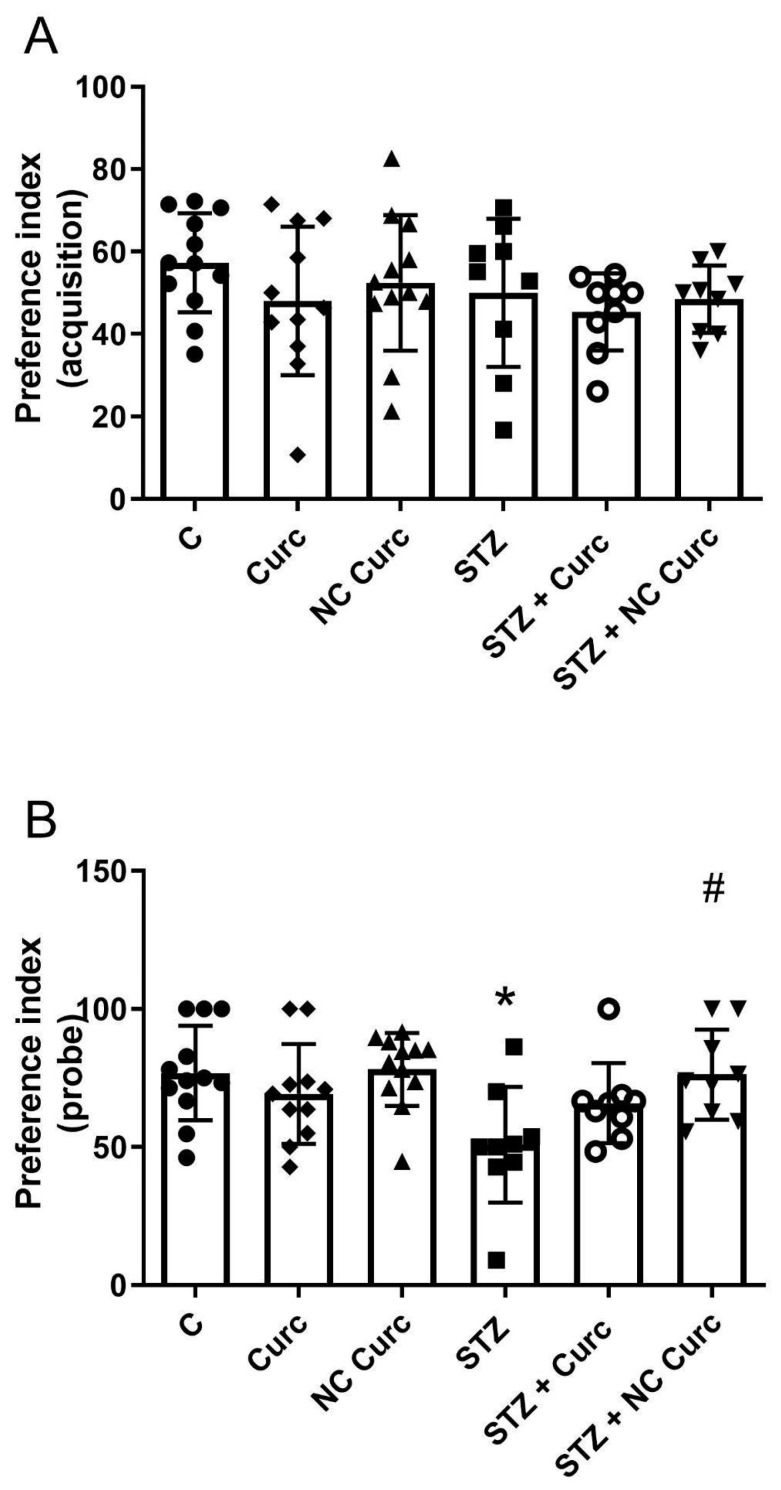
The NC Curc treatment restored the long-term memory impairment induced by STZ (icv) in rats in the ORT. Effects of NC Curc or curcumin treatments at a dose of 6 mg/kg/day on (**A**) exploratory preference during the (**A**) training and (**B**) LTM sessions assessed in the object recognition test in rats exposed to STZ (icv). Data are reported as the mean ± SD of 9–12 animals per group. * *p* < 0.05 compared to the control group, and # denotes *p* < 0.05 compared to the STZ group. ⬤ Denotes as Control; ◆ as Curc; ▲ as NC Curc; ◼ as STZ; ◯ as STZ + Curc and; ▼ as STZ + NC Curc group.

In the open field test, one-way ANOVA showed no significant difference in crossing numbers [F(5, 56) = 0.8161; *p* = 0.5433] or rearings [F(5, 56) = 0.5394; *p* = 0.7455] among the groups (Table 1). Figure 2 presents the effects of NC Curc on STZ-induced memory loss in the ORT. On day 33, all the animals explored both objects equally (about 50%) during the training session (Figure 2A) [F(5, 56) = 0.9068; *p* = 0.4833]. Significant differences in exploratory preference were observed in the probe ORT [F(5, 56) = 3.672; *p* = 0.0061] (Figure 2B). The STZ group exhibited a notably lower preference for the new object compared to the control group (*p* = 0.0108). The NC Curc treatment significantly restored the mnemonic function disrupted by STZ (*p* = 0.0251), indicating that NC Curc mitigated the STZ-caused LTM deficits in rats.

**Figure 3:**
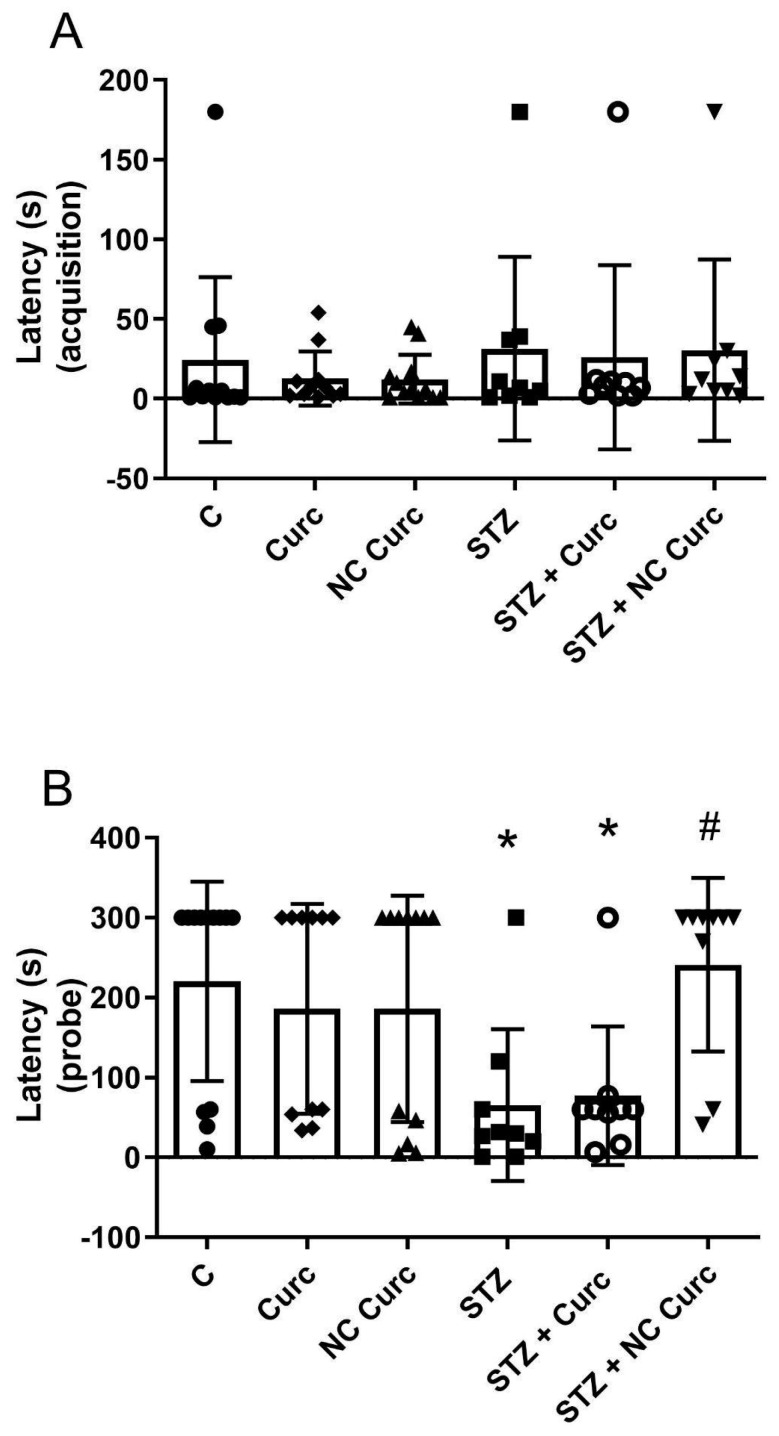
The NC Curc treatment restored the aversive memory impairment induced by STZ (icv) in rats in the inhibitory avoidance test. The effects of NC Curc or curcumin treatments (6 mg/kg/day) on (**A**) training (latency in seconds to fall from the platform) and (**B**) probe (latency in seconds to fall from the platform) in the inhibitory avoidance test in rats exposed to STZ (icv). Data are reported as the mean ± SD of 9−12 animals per group. * *p* < 0.05 compared to the control group, and # indicates *p* < 0.05 compared to the STZ group. ⬤ Denotes as Control; ◆ as Curc; ▲ as NC Curc; ◼ as STZ; ◯ as STZ + Curc and; ▼ as STZ + NC Curc group.

In the Y-maze test, no significant differences were found in the total arm entries [F(5, 56) = 1.125; *p* = 0.3580], suggesting similar locomotor activity across groups (Table 1). Analysis of actual alternation percentages also showed no significant differences [F(5, 56) = 1.407; *p* = 0.2360] (Table 1), indicating no effect of treatments on working memory. During inhibitory avoidance training, transfer latency times did not differ significantly between the groups [F(5, 56) = 0.4089; *p* = 0.8406] (Figure 3A). However, significant differences emerged in the probe test [F(5, 56) = 3.672; *p* = 0.0060] (Figure 3B), with STZ significantly reducing transfer latency compared to the control group (*p* = 0.0437). Notably, NC Curc reversed this impairment (*p* = 0.0312), aligning the STZ + NC Curc group with the control group. This suggests that NC Curc restored the aversive LTM impairment induced by STZ.

### 3.2. Oxidative Stress Markers

**Figure 4:**
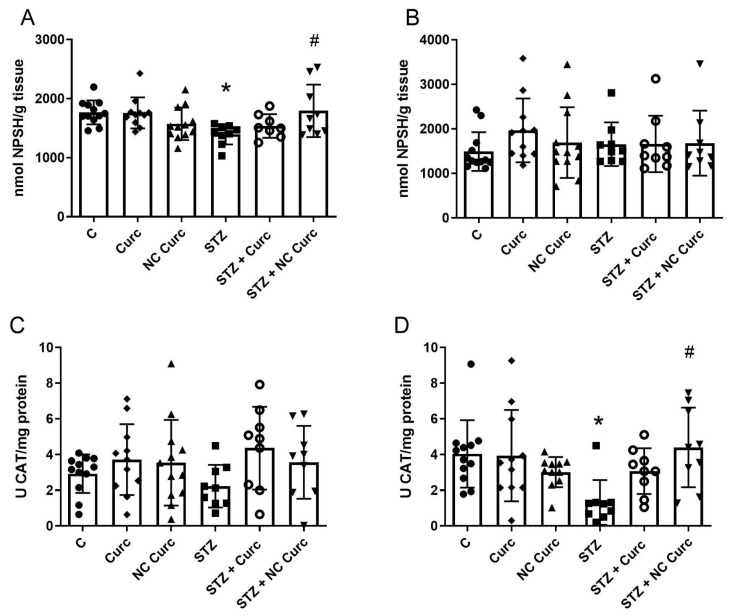
NC Curc treatment restores the depletion of NPSH levels and the inhibition of CAT activity in different cerebral structures of rats exposed to STZ (icv). Effects of NC Curc or curcumin treatments at a dose of 6 mg/kg/day on NPSH levels in the (**A**) prefrontal cortex and (**B**) hippocampus, as well as CAT activity in the (**C**) prefrontal cortex and (**D**) hippocampus of rats exposed to STZ. Data are presented as the mean ± SD of 9–12 animals per group. * *p* < 0.05 compared to the control group, and # indicates *p* < 0.05 compared to the STZ group. ⬤ Denotes as Control; ◆ as Curc; ▲ as NC Curc; ◼ as STZ; ◯ as STZ + Curc and; ▼ as STZ + NC Curc group.

No significant alterations in RS levels were observed in the prefrontal cortex [F(5, 56) = 0.03074, *p* = 0.9995] or hippocampus [F(5, 56) = 0.2276, *p* = 0.9489] following STZ or NC Curc/curcumin treatments (Table 2). TBARS levels showed no significant differences across the groups in either the prefrontal cortex [F(5, 56) = 0.5057, *p* = 0.7695] or hippocampus [F(5, 56) = 0.1363, *p* = 0.9825] (Table 2). STZ significantly decreased NPSH levels in the prefrontal cortex compared to the control group (*p* = 0.0384) (Figure 4A), but NC Curc effectively countered this reduction (*p* = 0.0382). No changes in NPSH levels were observed in the hippocampus across the groups [F(5, 56) = 0.5983, *p* = 0.7016] (Figure 4B). CAT activity in the prefrontal cortex showed no significant differences [F(5, 56) = 1.181, *p* = 0.3313] (Figure 4C), but a notable inhibition of hippocampal CAT activity was induced by STZ [F(5, 56) = 1.181, *p* = 0.3313], which was restored by NC Curc treatment (*p* = 0.0139) (Figure 4D).

### 3.3. Acetylcholinesterase Activity

**Figure 5:**
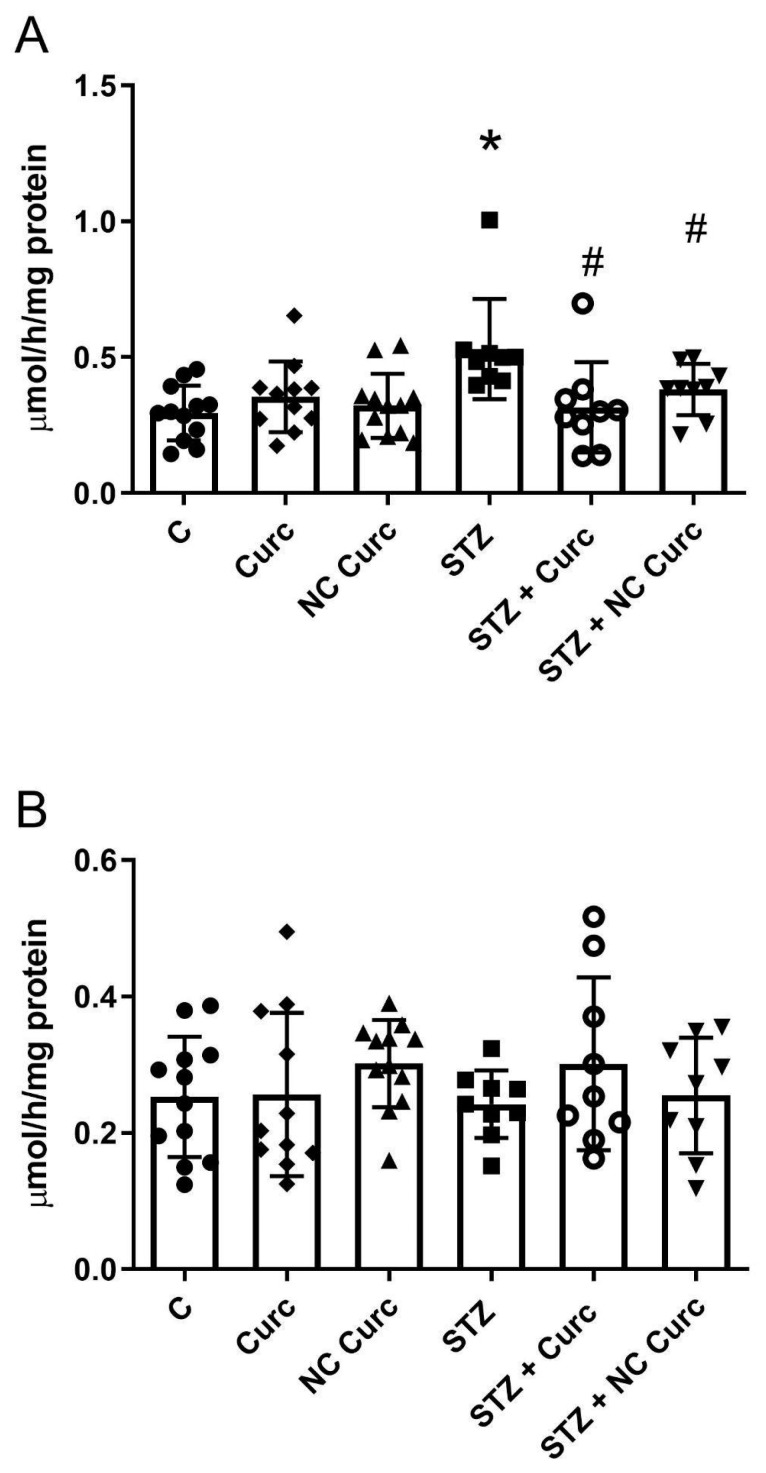
Both NC Curc and curcumin treatments inhibited the AChE activity enhanced by STZ in the rat prefrontal cortex. Effects of NC Curc (6 mg/kg/day) or curcumin (6 mg/kg/day) treatments on AChE activity in the (**A**) prefrontal cortex and (**B**) hippocampus of rats exposed to STZ. Data reported as mean ± SD of 9–12 animals/group. * *p* < 0.05 compared to control group, # indicates *p* < 0.05 compared to STZ group. ⬤ Denotes as Control; ◆ as Curc; ▲ as NC Curc; ◼ as STZ; ◯ as STZ + Curc and; ▼ as STZ + NC Curc group.

In the prefrontal cortex, AChE activity was significantly higher in STZ (icv)-induced rats compared to controls (*p* = 0.0040). Both NC Curc (*p* = 0.0146) and Curc treatments (*p* = 0.0176) markedly inhibited AChE activity in comparison to STZ icv-induced rats (Figure 5A). However, no changes in hippocampal AChE activity were observed across the treated groups [F(5, 56) = 0.8062, *p* = 0.5502] (Figure 5B).

### 3.4. Neuroinflamation Marker

**Figure 6:**
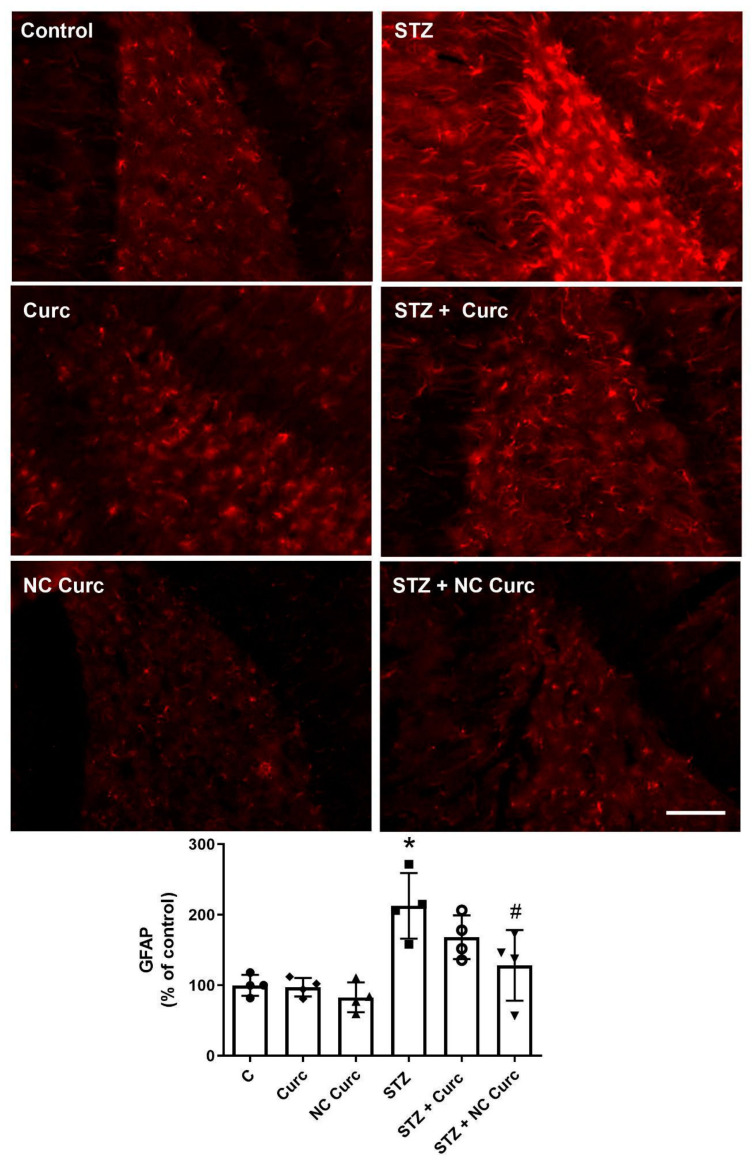
NC Curc treatment restored the GFAP content activated by STZ in the cerebral structures of rats. Effects of NC Curc (6 mg/kg/day) or curcumin (6 mg/kg/day) treatments on GFAP content in the cerebral structures of rats exposed to STZ. Representative image of GFAP content obtained by immunofluorescence microscopy of the dentate gyrus region. Scale bar: 100 μm. Data from quantitative analyses are reported as mean ± SD of 4 animals/group. * *p* < 0.05 compared to the control group, # indicates *p* < 0.05 compared to the STZ group. ⬤ Denotes as Control; ◆ as Curc; ▲ as NC Curc; ◼ as STZ; ◯ as STZ + Curc and; ▼ as STZ + NC Curc group.

The analysis of GFAP content showed significant differences between the groups [F(5, 18) = 9.381, *p* = 0.0002], as depicted in Figure 6. The STZ group exhibited increased GFAP levels compared to the control group (*p* = 0.0014). NC Curc treatment effectively restored GFAP content to levels comparable to the control group (*p* = 0.0188).

## 4. Discussion

This study demonstrated that bilateral administration of STZ (icv) resulted in memory impairment, as indicated by changes in behavior in the ORT and inhibitory avoidance, as well as increased oxidative stress and activation of AChE and GFAP. Importantly, our findings revealed that NC Curc reversed the memory impairment and other changes caused by STZ. The therapeutic effect of the nanoformulation appears to be more effective than conventional curcumin treatment.

In behavioral terms, the initial two weeks following STZ administration were characterized by progressive and enduring deficits in learning and memory, which persisted for up to 12 weeks after infusion [35]. Numerous studies have shown that memory impairments become well-established 21 days after STZ administration [2,3,16,36]. Within this timeframe, deficits in rodent memory have consistently been observed through tests such as the ORT [36], the inhibitory avoidance test [37], and the Y-maze test [3]. Therefore, we intentionally waited for 21 days before starting the therapies involving curcumin and NC Curc, as our main objective was to assess the therapeutic effects of these formulations in mitigating the established memory deficits.

In this study, STZ induced a decline in LTM in the ORT, where the animals did not show a preference for the novel object, similar to the sporadic type of AD [3,36,38,39,40]. Furthermore, STZ impaired memory retention in the inhibitory avoidance test, which is consistent with a similar model reported by Gerzson et al. [37] and Pinz et al. [39]. Importantly, only treatment with NC Curc reversed the memory impairment caused by STZ in both the ORT and the inhibitory avoidance test, leading us to conclude that the nanoformulation containing curcumin enhances the effects of curcumin. The absence of changes in locomotor activity aligned with the finding of the restoration of memory impaired by STZ through NC Curc, indicating that this effect is indeed mnemonic.

Huang and colleagues [41] demonstrated the effectiveness of PLGA nanoparticles, modified with a blood–brain barrier-penetrating peptide and loaded with curcumin, in alleviating memory deficits in a double transgenic AD model (APP/PS1dE9) during the ORT in mice. Additionally, de Carvalho and colleagues [42] showed that NC Curc was more potent than curcumin in inhibiting the angiogenic process in a chick embryo model, which they attributed to the process of release and absorption of the bioactive compound and the surfactant properties of the P80 coating of this nanocapsule, which enhances membrane permeation. Our research group also highlighted the curcumin nanocapsules’ higher efficacy than free curcumin in protecting against oxidative alterations and depressive-like behavior induced by β-amyloid administration in mice [13]. Thus, our findings support these studies, as NC Curc improved different types of memory in the AD models. The enhanced penetration capacity, along with the optimized process of release and absorption of NC, could explain the effectiveness of the nanomaterial in reversing memory impairment caused by STZ.

Our results suggest that oxidative stress is involved in the effects of STZ, and treatment with NC Curc reverses these effects. It has been shown that oxidative stress in DA leads to changes in the cell signaling pathway, resulting in neuroinflammation [43,44,45]. However, we did not observe significant differences in ROS and TBARS levels in any of the analyzed structures. This lack of distinction may be due to the sensitivity of both techniques, which could have influenced our results, or limitations in the STZ model, as observed by Rodrigues and colleagues [46]. It is also important to acknowledge the limitations inherent in the chosen model. Singh and Kumar [43], in a comparative study of AD induction models, observed that animals treated with intrahippocampal Aβ (1–42) exhibited greater oxidative damage compared to those treated with STZ (icv).

Conversely, our results revealed decreased cortical levels of NPSH and hippocampal activity of CAT induced by STZ. However, both of these effects were increased by NC Curc treatment. Similar effects were observed in a neuroprotective study conducted by Rodrigues and colleagues. They attributed these effects to the antioxidant properties of NC Curc, which result from its prolonged and sustained action due to nanoformulation [47].

As a non-enzymatic antioxidant defense, NPSH levels play a fundamental role in detoxification reactions. The decrease in GSH levels in postmortem human brain samples with age suggests their potential involvement in the development of AD [48]. Additionally, a deficiency or malfunction of CAT, a crucial antioxidant enzyme that converts hydrogen peroxide to water and oxygen, appears to be associated with diseases like AD. The current theory on how β-amyloid induces oxidative damage in cells proposes a direct interaction with catalase, which deactivates the protein’s catalytic activity, leading to the onset of oxidative stress in AD [49]. While changes in antioxidant defenses have been observed, further experiments, including evaluating antioxidant enzyme expression, are necessary for a comprehensive understanding. Robust validation is crucial to support significant claims about the antioxidant mechanism, prompting caution when interpreting our findings. Thus, future investigations are vital to confirm and strengthen the identified hypothesis of the antioxidant effect of NC Curc.

In line with previous studies [15,17], it has been found that STZ increases AChE activity in the cortex. It is well known that cholinergic neurotransmission plays a crucial role in AD, and anticholinesterases have been proven effective in treating the disease and improving cognitive function in patients [50]. Therefore, the dysregulation of AChE is a characteristic of AD and is associated with the neurobiological processes involved in memory and cognition. In a study conducted on rats, it was observed that those treated with STZ exhibited a notable rise in AChE activity in the cortex, suggesting cholinergic dysfunction. This finding aligns with previous reports [43], although not in the hippocampus.

In our study, we found that both curcumin and NC Curc treatments were effective in restoring the increased AChE activity caused by STZ in the prefrontal cortex of rats. Noor and colleagues also observed similar effects with curcumin loaded in nanoparticles, which disrupted AChE activity. The antioxidant properties of NC Curc were found to be correlated with its effectiveness [15]. The variation in responses to oxidative stress or AChE disruption in different brain structures is influenced by several factors, including functional characteristics, lipid composition, neuronal density, metabolic demands, unique antioxidant capacity, and the expression of cholinesterases specific to each region of the brain [51,52]. These factors help explain the differences observed among structures in our study. It is worth noting that curcumin and its derivatives have shown moderate inhibitory potential on AChE, which contributes to their role in restoring memory [52,53,54,55,56].

The excessive activation of astrocytes, as indicated by the increased expression of GFAP [57], is a pathological sign of neurodegenerative damage. The STZ group showed a significant increase in astrocyte activation, consistent with the AD model and other studies [1,17,58,59]. Astrocytes play a critical role in maintaining homeostasis, including protecting neurons against oxidative stress and promoting neuroinflammation. In our study, we discovered that NC Curc could reverse the elevated levels of GFAP caused by STZ. This finding aligns with previous research demonstrating the strong anti-inflammatory properties of curcumin. Hoppe and colleagues [60] also observed a decrease in GFAP levels with NC Curc treatment, attributing it to the improved bioavailability of curcumin in the brain, significantly reducing neuroinflammatory processes. Consistent with these findings, STZ (icv) administration induced oxidative stress and alterations in AChE activity associated with neuroinflammation. It is worth noting that among the treatments given to the rats, NC Curc exhibited the highest effectiveness in reversing the effects of this AD model.

## 5. Conclusions

Our findings suggest that NC Curc has the potential to restore memory and reverse structural changes in the cortex and hippocampus of rats induced by STZ icv. This therapeutic effect is likely due to the antioxidant and anti-inflammatory properties of curcumin, which can be enhanced through nanoencapsulation. The observed improvements in memory behaviors, reduced oxidative stress levels, normalized AChE activity, and amelioration of neuroinflammatory markers collectively indicate the restorative potential of NC Curc in brain regions affected by STZ-induced damage.

Our study represents a significant advancement in understanding the effects of NC Curc in Alzheimer disease. The experimental design specifically focused on exploring these nanotechnological formulations’ therapeutic and neurorestorative effects. Further investigations will be needed in the future to determine its viability as a therapeutic agent for Alzheimer disease.

## Figures and Tables

**Table 1:** The groups did not differ regarding the open field and Y-maze tests.

Open Field Test			Y-Maze Test	
	Crossing	Rearing	Arm Entries	Alternations
Control	48.38 ± 24.38	25.54 ± 18.18	14.45 ± 5.02	53.73 ± 20.30
Curc	46.50 ± 23.03	21.55 ± 12.50	13.39 ± 3.96	45.18 ± 14.86
NC Curc	46.00 ± 28.64	23.58 ± 18.00	13.25 ± 4.71	50.67 ± 19.66
STZ	48.72 ± 23.19	27.28 ± 16.38	16.89 ± 5.77	52.46 ± 12.76
STZ + Curc	57.78 ± 28.64	20.33 ± 9.77	16.13 ± 5.90	37.63 ± 8.18
STZ + NC Curc	34.19 ± 25.52	17.06 ± 14.79	12.75 ± 3.73	52.23 ± 14.74

The effects of NC Curc and curcumin treatments (6 mg/kg/day) on total crossing and rearing in the open field test and number of arm entries and % alternation in the Y-maze test after intracerebroventricular STZ administration in rats are shown in Table 1. Data are reported as the mean ± SD of 9–12 animals per group.

**Table 2:** The groups did not differ in ROS and TBARS levels as markers of oxidative stress.

RS			TBARS	
	Prefrontal Cortex	Hippocampus	Prefrontal Cortex	Hippocampus
Control	287.4 ± 193	498.5 ± 304	1.257 ± 0.28	1.211 ± 0.31
Curc	272.6 ± 235	546.5 ± 206	1.345 ± 0.52	1.125 ± 0.35
NC Curc	368.6 ± 335	423.8 ± 265	1.175 ± 0.39	1.128 ± 0.29
STZ	267.5 ± 144	516.1 ± 276	1.123 ± 0.33	0.954 ± 0.31
STZ + Curc	270.4 ± 158	485.9 ± 269	1.205 ± 0.65	1.359 ± 0.37
STZ + NC Curc	298.6 ± 180	464.2 ± 382	1.370 ± 0.42	1.076 ± 0.31

Effects of NC Curc and curcumin treatments at 6 mg/kg/day on TBARS and RS levels in the prefrontal cortex and hippocampus of rats exposed to STZ. Malondialdehyde levels were expressed as nmol MDA/mg protein, and RS results were expressed as units of fluorescence (UF). Data are reported as the mean ± SD of 9–12 animals per group.

## Data Availability

The complete dataset supporting the obtained results is included within the article, and there is no need for supplementary source data.
